# Identification Method for Railway Rail Corrugation Utilizing CEEMDAN-PE-SPWVD

**DOI:** 10.3390/s24248058

**Published:** 2024-12-17

**Authors:** Jianhua Liu, Kexin Zhang, Zhongmei Wang

**Affiliations:** College of Railway Transportation, Hunan University of Technology, Zhuzhou 412007, China; jhliu@hut.edu.cn (J.L.); kexin_hut@163.com (K.Z.)

**Keywords:** rail corrugation identification, complete ensemble empirical mode decomposition, smoothed pseudo Wigner–Ville distribution, time–frequency characteristic analysis

## Abstract

Rail corrugation intensifies wheel–rail vibrations, often leading to damage in vehicle–track system components within affected sections. This paper proposes a novel method for identifying rail corrugation, which combines Complete Ensemble Empirical Mode Decomposition with Adaptive Noise (CEEMDAN), permutation entropy (PE), and Smoothed Pseudo Wigner–Ville Distribution (SPWVD). Initially, vertical acceleration data from the axle box are decomposed using CEEMDAN to extract intrinsic mode functions (IMFs) with distinct frequencies. PE is used to evaluate the randomness of each IMF component, discarding those with high permutation entropy values. Subsequently, correlation analysis is performed on the retained IMFs to identify the component most strongly correlated with the original signal. The selected component is subjected to SPWVD time–frequency analysis to identify the location and wavelength of the corrugation occurrence. Filtering is applied to the IMF based on the frequency concentration observed in the time–frequency analysis results. Then, frequency–domain integration is performed to estimate the rail’s corrugation depth. Finally, the algorithm is validated and analyzed using both simulated data and measured data. Validation results show that this approach reliably identifies the wavelength and depth characteristics of rail corrugation. Additionally, the time–frequency analysis results reveal variations in the severity of corrugation damage at different locations.

## 1. Introduction

As railway transportation demand increases, prolonged use of track infrastructure has led to frequent wave–like wear (corrugation) on rail surfaces, characterized by periodic wear patterns along the rail length [1,2]. When trains pass over corrugated sections, high-frequency impact vibrations between the vehicle and the track induce abnormal wheel–rail forces, causing fatigue damage to components such as vehicles, rails, and fasteners, thereby complicating maintenance and repair [3,4]. In response to these challenges, this paper conducts a comprehensive investigation into methods for identifying rail corrugation under railway transportation conditions, which is essential for ensuring safe vehicle operation and effective maintenance of rail irregularities.

The most common method for identifying rail corrugation irregularities involves directly measuring the rail surface. However, this approach significantly increases staff workload [5]. Therefore, it is crucial to find an easily measurable and effective indicator supported by efficient data processing methods to identify rail surface irregularities. A review of the existing literature shows that the primary methods for identifying rail corrugation irregularities involve analyzing vehicle system dynamics indicators, such as wheel–rail vertical force and axle box vertical acceleration, using various data processing techniques [6]. Zhai et al. [7] simulated bogie acceleration data using SIMPACK 2021 software, diagnosing and identifying rail corrugation through continuous wavelet time–frequency analysis and eigenmodes obtained from empirical mode decomposition. Zilong Wei et al. [8] collected vehicle internal vibration acceleration responses when trains passed over rail corrugation damage, identifying the joint time–frequency distribution of internal noise at various train velocities and track radii. They proposed an indirect method based on internal noise to detect high-speed rail corrugation wavelengths, which can identify the location and wavelength of rail corrugation at different train speeds. Lang et al. [9] decomposed simulated axle box vibration acceleration signals using wavelet packet decomposition. By comparing the wavelet packet energy entropy of vibration signals across different nodes, they extracted energy distribution characteristics from the signals, effectively identifying single-wavelength rail corrugation features. Despite these advances, analyses of train dynamic responses under rail corrugation disturbances remain limited, and current methods often fail to align rigorously with actual operating conditions during validation.

Ensemble Empirical Mode Decomposition (EEMD), proposed by Huang and Wu [10], is an effective method for processing nonlinear and non-stationary signals developed in recent years. However, the EEMD algorithm relies on a limited number of ensemble averages to eliminate white noise interference, often failing to completely remove such interference, which results in distortion in the reconstructed signals. Complete Ensemble Empirical Mode Decomposition with Adaptive Noise (CEEMDAN), an improved version of EEMD, was introduced by Torres et al. [11]. This method introduces targeted noise at each decomposition stage, replacing Gaussian white noise. It not only overcomes the limitations of EEMD, but also improves the completeness of the decomposition process. Numerical simulations by Lv et al. [12] show that CEEMDAN effectively mitigates mode mixing and produces accurate intrinsic mode functions (IMFs). Since the IMFs obtained through CEEMDAN represent the natural oscillation modes embedded within the signal [13], extracting their permutation entropy (PE) as a feature matrix reveals the local multi-scale characteristics of vibration signals. This approach provides a more comprehensive criterion for mode screening.

Time–frequency analysis methods are effective in extracting the characteristics of frequency components in digital signals, as they vary across time and space. The Wigner–Ville Distribution (WVD) performs exceptionally well in extracting the time–frequency characteristics of single-component signals, offering optimal time–frequency concentration. However, as a typical quadratic transform, WVD is prone to cross-interference terms when analyzing multi-component signals. To mitigate these issues, the Smoothed Pseudo Wigner–Ville Distribution (SPWVD) was developed. The SPWVD method uses time domain and frequency domain window functions, applying convolution between the WVD and smoothing windows to effectively suppress cross-interference [14]. Chen et al. [15] applied the SPWVD transformation to bearing vibration data, compressed the resulting time–frequency images, and used them as input for a CNN. The experimental results demonstrated an improvement in the average classification accuracy of bearing fault data.

To sum up, this paper introduces a novel method for identifying rail corrugation using CEEMDAN, PE, and SPWVD techniques. Initially, CEEMDAN is used to decompose axle box vibration acceleration signals into IMFs with distinct frequency and amplitude characteristics. PE is applied to evaluate the randomness of each IMF component, discarding those with high entropy values to minimize noise interference in signal analysis. A correlation analysis is subsequently conducted on the remaining components to select the IMF most correlated with the original signal. This component undergoes SPWVD analysis to extract its time–frequency characteristics, enabling the determination of corrugation location and wavelength. Based on the frequency concentration observed in the time–frequency analysis, filtering is applied to the IMF component, followed by frequency domain integration to estimate corrugation depth. Finally, the method was verified and analyzed using simulated data from a 3-D wheel–rail finite element model alongside actual measured data.

## 2. Railway Rail Corrugation Wavelength Identification Model

### 2.1. Model Architecture

**Figure 1:**
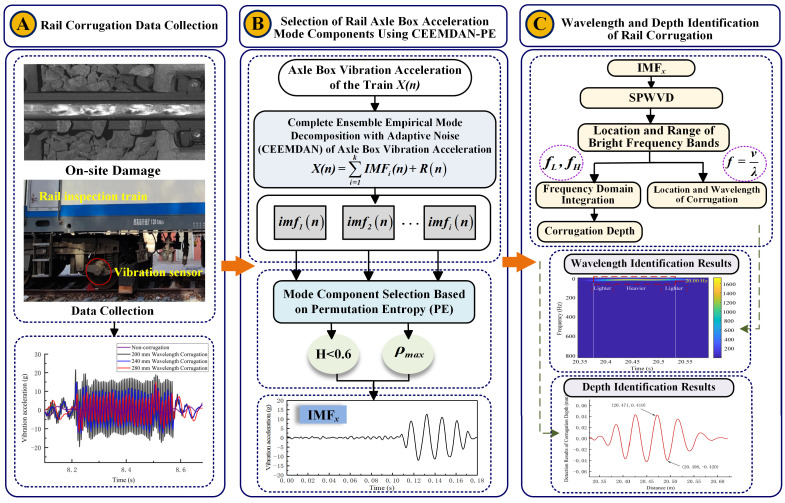
Overall Architecture Diagram.

The process architecture of the CEEMDAN-PE-SPWVD-based rail corrugation identification method is shown in Figure 1. Module A represents collecting vibration acceleration data from a specific railway line in China, providing the data foundation for validating the proposed algorithm. Module B represents the selection of vibration response components induced solely by corrugation using the CEEMDAN-PE method. Initially, CEEMDAN is used to decompose the vibration acceleration data and extract different signal components. As decomposition results contain pseudo-components and non-corrugation-induced signals, PE analysis is applied to each component to exclude highly non-stationary IMFs. Correlation analysis then selects the component *IMF_x_* with the highest correlation to the original signal. In Module C, SPWVD time–frequency analysis is applied to the selected *IMF_x_* to extract its time–frequency characteristics. The location of bright frequency bands and their central frequencies enable the determining of corrugation location and wavelength. Subsequently, *IMF_x_* undergoes mixed filtering, with the cutoff frequency set according to the frequency band range of the bright frequency band. The filtered signal is then integrated into the frequency domain to obtain the rail corrugation depth.

### 2.2. Selection of Axle Box Acceleration Signal Mode Component Using CEEMDAN-PE

To accurately identify the wavelength of rail corrugation, it is essential to isolate the vibration response induced by rail corrugation while minimizing pseudo-components and noise in the signal. This study employs the CEEMDAN method to extract various components of the signal. CEEMDAN decomposes the signal into multiple IMFs, each representing a distinct frequency component [12,16]. The decomposition process of this algorithm is as follows [17]:

1. Let V(n) denote the original vertical axle box acceleration signal. Add Gaussian white noise $W^{i}(t)$ that follows a normal distribution N(0,1); the first-order component of CEEMDAN is shown in Equation (1):
  (1) $$\bar{IMF_{1}}(n)=\frac{1}{I}\sum_{i=1}^{I}IMF_{1}^{i}\left(n\right)$$
  where I represents the total number of noise additions, and i denotes the current iteration of noise addition.
2. Construct the next decomposed signal as follows: $V\left(n\right)=V\left(n\right)+\eta_{i}W^{i}\left(n\right)$, resulting in ${\mathrm{IMF}}_{2}$.
3. Repeat the first two steps until completion. The final residual term is shown in Equation (2).

(2) $$R\left(n\right)=V(n)-\sum_{i=1}^{k}IMF_{i}(n)$$

In the equation, k represents the number of generated IMFs.

The CEEMDAN decomposition results reveal that the original signal contains pseudo-components [18] introduced by noise and irregular signals unrelated to corrugation, potentially impacting subsequent analysis [19]. To enhance analytical accuracy, the study employs the PE method [20] to assess the randomness of each IMF. By calculating the permutation entropy H of each IMF component, highly random components are excluded, thereby reducing interference in signal analysis. The process is outlined as follows [16]:

Let a time series be denoted as $Y=\left\{y\left(i\right),\;i=1,2,\ldots ,k\right\}$. Given a dimension m, reconstruct the time series and perform ascending sorting on the elements in the reconstructed matrix. There are a total of m! possible permutation orders, with each element sequence having an occurrence probability denoted by $p_{1},p_{2},\ldots \;,p_{q}$.

The permutation entropy of the time series Y is calculated as [21]:

(3) $$H\left(m\right)=-\sum_{k=1}^{q}P_{k}\ln P_{k}$$

After removing high-randomness IMF components based on the set permutation entropy threshold e, the mode component with the highest correlation coefficient, *IMF_x_*, is selected as the representative component that most accurately reflects the characteristics of the original signal. The formula for calculating the correlation coefficient is given below [22]:

(4) $$\rho =\frac{\sum_{m=0}^{\infty }x(m){\mathrm{IMF}}_{i}}{{\left[\sum_{m=0}^{\infty }x^{2}\left(m\right)\sum_{m=0}^{\infty }{\mathrm{IMF}}_{i}^{2}\right]}^{\frac{1}{2}}}$$

In the equation, x(m) represents the original signal and ${\mathrm{IMF}}_{i}$ is the i-th IMF component, respectively.

### 2.3. Identification of Rail Corrugation Wavelength and Depth Using SPWVD Time–Frequency Analysis

The WVD, introduced by Wigner [23] and Ville, tends to produce cross-interference terms when analyzing multi-component signals in the time–frequency domain, obscuring the energy distribution among signal components. To achieve high time–frequency resolution and ensure energy positivity while mitigating the effects of cross-interference terms in the WVD, this study employs the SPWVD for analyzing non-stationary axle box acceleration signals. The SPWVD of a signal is expressed as [24]:

(5) $$SPWVD_{g,h}(t,f)=\iint g(u)h(\tau )x(t-u+\tau /2)x^{*}(t-u-\tau /2)e^{2j\pi f\tau }dud\tau$$

where $g\left(u\right)$ and $h\left(\tau \right)$ denote the window functions in the frequency and time domains, respectively.

The distribution of bright frequency bands in the SPWVD time–frequency analysis results allows for precise localization of rail corrugation fault positions in the time domain. Additionally, the central frequency and energy amplitude of these bands enable the determination of the corrugation passing frequency and its severity at different positions along the rail. Using the relationship between corrugation wavelength, passing frequency, and vehicle speed, the wavelength of corrugation can be calculated, as shown in Equation (6):

(6) $$f=\frac{v}{\lambda }$$

The vertical vibration acceleration signal of the axle box theoretically reflects the geometric profile of the rail surface based on the relationship between acceleration and displacement. To minimize integration errors, a frequency domain integration method utilizing the Fast Fourier Transform (FFT) is applied to derive the corrugation depth characteristics of the rail [25]. Since the original signal contains pseudo-components and noise, direct filtering may yield a complex frequency composition. Therefore, this study analyzes the corrugation vibration response component selected through the CEEMDAN-PE method. The filtering frequency range $\left[f_{L},f_{H}\right]$ is then determined based on the bright frequency band range identified in the SPWVD time–frequency analysis of this component. The main steps are outlined as follows:

1. Based on the above depth identification method, process the vertical vibration acceleration data x(n) of the axle box to determine the frequency range $\left[f_{L},f_{H}\right]$ for filtering. Perform bandpass filtering within this range, and denote the filtered data as $x^{\prime }(n)$.
2. Use $x^{\prime }(n)$ as the input to calculate the Fourier Transform for displacement X(ω):
  (7) $$X\left(\omega \right)=-\frac{1}{\omega^{2}}\left[A\left(\omega \right)+n\omega h_{0}+v_{0}\right]$$

In the equation, ω represents the angular frequency; A(ω) is the Fourier Transform of $x^{\prime }\left(n\right)$; and $h_{0}$ and $v_{0}$ are the initial values of vertical displacement and velocity, respectively.

3. Calculate the corrugation depth of the rail as follows:

(8) $$h=F^{-1}\left[X\left(\omega \right)\right]$$

In the equation, $F^{-1}$ denotes the inverse Fast Fourier Transform.

## 3. Methods Validation and Analysis

### 3.1. Vibration Signal Simulation and Acquisition

#### 3.1.1. Simulation and Validation Based on ABAQUS Wheel–Rail Coupled Model

Due to the limited range of corrugation wavelengths obtainable from measured axle box vibration acceleration data, a 3-D contact finite element model of the wheel–rail system is developed using ABAQUS 2021 software to validate the algorithm’s generality [26]. This model simulates the dynamic behavior of the wheel–rail system as a train traverses rails with different corrugation wavelengths. The simulation data derived from this model are employed to verify the proposed algorithm.

**Figure 2:**
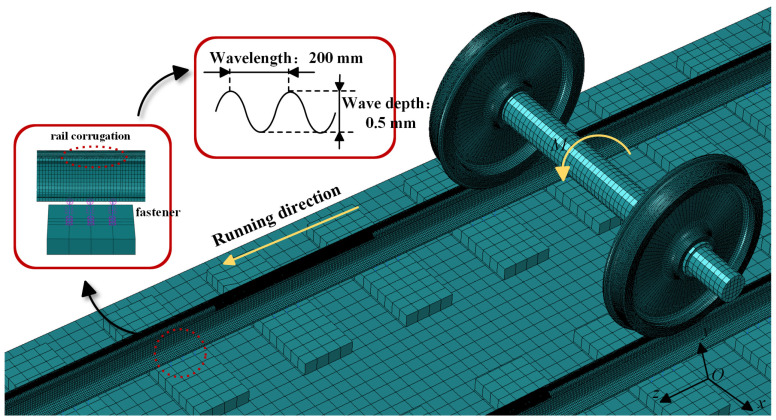
Finite element simulation model.

This study employs measured parameters from China’s C80 heavy-haul trains to develop a structural model of the vehicle–track system. The model comprises components such as the wheelset, smooth rail, corrugated rail, fastening device, and track slab. In the vehicle system, the primary suspension is modeled using Spring A to simplify the model while accurately simulating the system’s dynamic characteristics [27]. The wheelsets, rails, and track slabs are modeled using three-dimensional Lagrangian solid elements [28]. The finite element simulation model is shown in Figure 2, and the calculation parameters and conditions are listed in Table 1.

**Figure 3:**
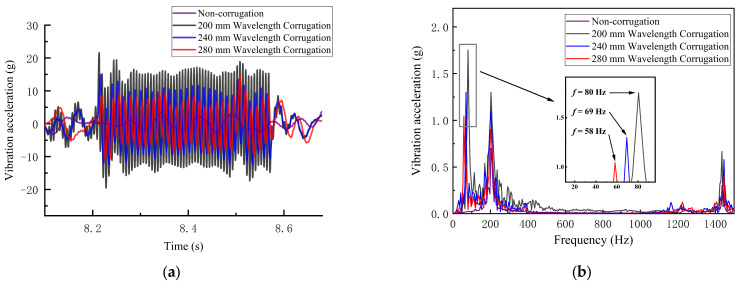
Simulation data illustrating various wavelengths of rail corrugation: (**a**) axle box vertical vibration acceleration—time history; (**b**) axle box vertical vibration acceleration—power spectral density.

Using the model developed in this study, we investigate the characteristics of the vertical vibration acceleration response of the axle box in the wheel–rail system disturbed by rail corrugation irregularities. According to the literature [3], the vibration frequency caused by corrugation in heavy-haul railway tracks typically ranges from 50 to 100 Hz. Therefore, this study analyzes rail corrugation wavelengths ranging from 200 to 300 mm. The corrugation depth of the rail is set to 0.5 mm. Figure 3 displays the vertical vibration acceleration response curves and their power spectral density for axle boxes disturbed by rail corrugation with wavelengths of 200 mm, 240 mm, and 280 mm at a travel speed of 60 km/h. The corrugation depth is 0.5 mm.

Figure 3a illustrates that as the wheelset enters the corrugated rail area, the vertical vibration acceleration of the axle box displays a distinct high-frequency vibration characteristic, with response amplitude diminishing as the corrugation wavelength increases. This high-frequency characteristic reflects the dynamic influence of rail corrugation on the vehicle–track system [29]. Further Fourier Transforms of the axle box vibration acceleration signal yield the results shown in Figure 3b. Analysis reveals that scenarios with and without corrugation excitation exhibit similar frequency components. In the low-frequency range of 0–1000 Hz, an energy concentration of 180 Hz is observed under both conditions. This analysis reveals that the scenarios with and without corrugation excitation share similar frequency components. However, energy concentration occurs only under corrugation conditions at frequency bands around 80 Hz, 69 Hz, and 58 Hz in Figure 3b, with the amplitude of axle box vibration acceleration decreasing in these bands as the wavelength increases. The corrugation passing frequencies calculated using Equation (6) are 83.33 Hz, 69.44 Hz, and 59.52 Hz, closely aligning with the time–frequency analysis results. This consistency suggests that finite element model simulation data can effectively analyze the impact of rail corrugation on the dynamic performance of the wheel–rail system, further supporting the feasibility of detecting rail corrugation based on axle box vibration acceleration.

#### 3.1.2. Acquisition of Measured Vibration Signals

**Figure 4:**
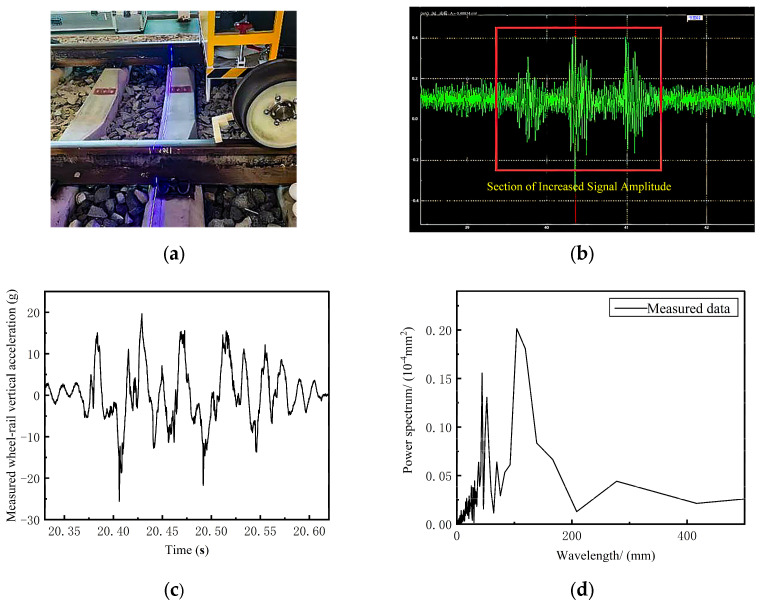
Acquisition and processing of measured signal: (**a**) schematic diagram of rail corrugation measurement; (**b**) channel display of vibration signal in the corrugation section; (**c**) vibration acceleration signal in the corrugation section; (**d**) power spectrum of rail surface straightness.

To demonstrate the effectiveness of the CEEMDAN-PE-SPWVD corrugation identification method in practical applications, this study conducted field measurements on a corrugated section of an actual railway line, collecting axle box vibration acceleration signals as the train passed through the section. The field setup is shown in Figure 4a, with a vehicle test speed of 10 km/h and a sampling frequency of 4 kHz. Figure 4b displays the real-time response of the axle box acceleration signal recorded during the train’s passage through the corrugated section. A segment of the signal with a large amplitude was selected, and 3000 data points were collected. Figure 4c shows the down-sampled and filtered result of this data segment. Figure 4d displays the power spectrum of rail surface straightness for this data segment, revealing that the power energy amplitude is concentrated in the wavelength range of 135 mm to 145 mm.

### 3.2. Experimental Validation and Analysis

#### 3.2.1. Method Validation Using Simulation Data

The study processed simulated vibration data from the wheel–rail finite element model for two scenarios: a 200 mm corrugated rail and a smooth rail without corrugation, following the algorithm workflow. The CEEMDAN algorithm’s parameters had minimal impact on the decomposition results for vertical axle box vibration acceleration within a specific range. In this study, parameters were configured based on CEEMDAN’s applications in scenarios such as bearing vibration signals [30], permanent magnet synchronous motor vibration signals [31], and blasting vibration signals [32]. The noise standard deviation was set to 0.2, the number of iterations to 100, and the maximum screening iterations to 300 [33], resulting in the decomposition of 11 IMFs with distinct frequencies. The permutation entropy value of each IMF was calculated, with a threshold set at 0.6 [34]; IMF components exceeding this threshold were discarded due to high randomness. A correlation coefficient threshold of 0.3 was also established; IMF components below this threshold were considered to have a low signal-to-noise ratio and were removed accordingly [35].

**Figure 5:**
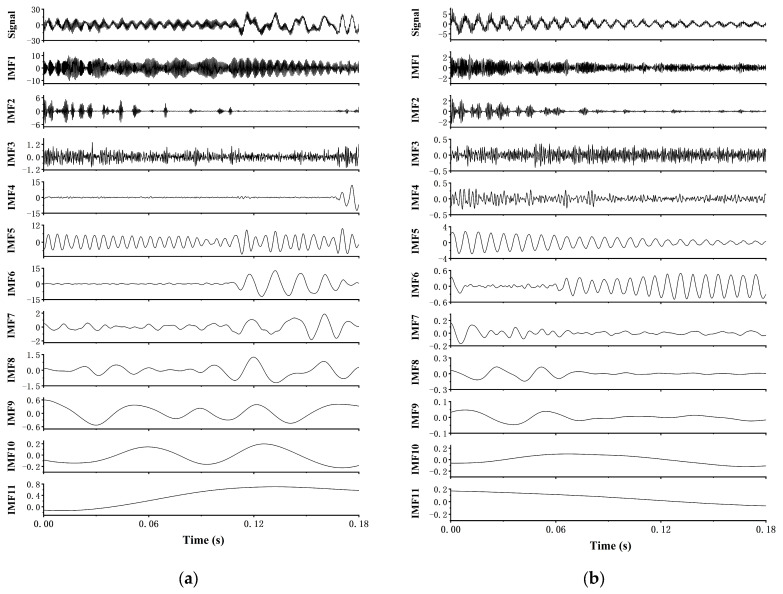
CEEMDAN decomposition of vibration acceleration signal: (**a**) 200 mm wavelength corrugation excitation; (**b**) no corrugation excitation.

**Figure 6:**
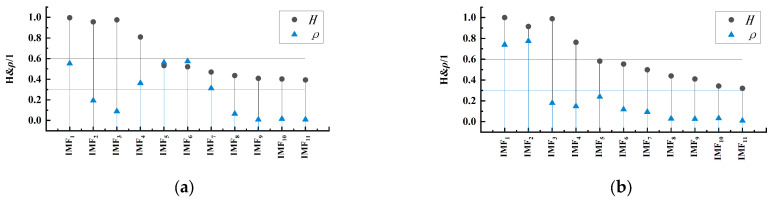
Distribution of PE Values and correlation coefficients: (**a**) 200 mm wavelength corrugation excitation; (**b**) no corrugation excitation.

The CEEMDAN decomposition results are shown in Figure 5. Overall, the complex original signal is effectively decomposed into components at different scales. To differentiate these components, further processing of the decomposition results is required. Figure 6 shows the distribution of Permutation Entropy values H and correlation coefficients for each IMF component. As shown in Figure 6b, under non-corrugated conditions, no IMF components passed the screening, indicating that the combination of CEEMDAN and PE analysis can be used to detect rail corrugation damage. Finally, correlation analysis was conducted on the decomposition results, selecting IMF6 as the optimal component. The waveform of this component exhibits regularity, primarily reflecting the vibration characteristics induced by rail corrugation.

**Figure 7:**
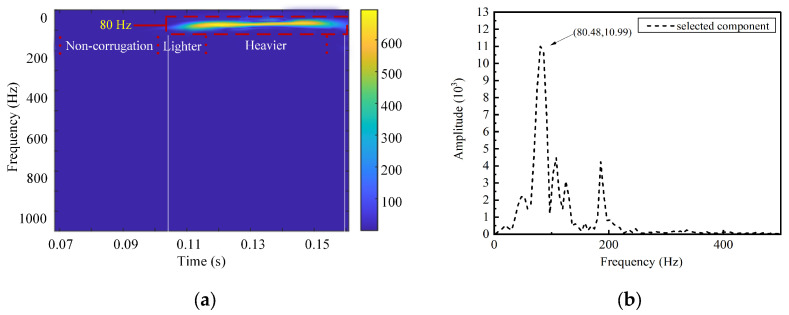
Time–frequency analysis for 200 mm wavelength corrugation: (**a**) SPWVD time–frequency representation; (**b**) Fourier Transform frequency spectrum.

**Figure 8:**
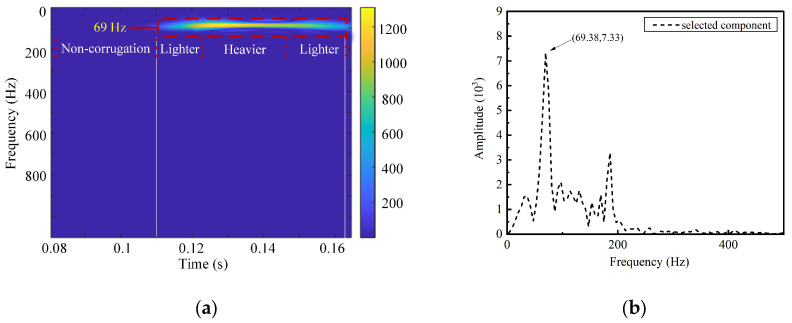
Time–Frequency Analysis for 240 mm Wavelength Corrugation: (**a**) SPWVD Time–Frequency Representation; (**b**) Fourier Transform Frequency Spectrum.

**Figure 9:**
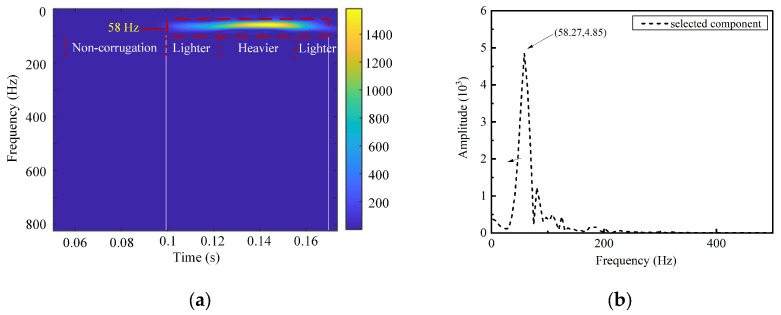
Time–frequency analysis for 280 mm wavelength corrugation: (**a**) SPWVD time–frequency representation; (**b**) Fourier Transform frequency spectrum.

A further analysis of the time–frequency characteristics of the IMF6 component is conducted using the SPWVD. The results are shown in Figure 7a. According to Equation (6), the characteristic frequency of a 200 mm wavelength corrugation is 83.3 Hz. The figure shows a prominent bright frequency band around 80 Hz, closely matching the characteristic frequency of rail corrugation. Figure 7b depicts the Fourier Transform outcomes of IMF6, indicating that the frequency of this component is relatively concentrated, with a central frequency of 80.48 Hz. The algorithm’s identification accuracy is calculated to be 96.57%. Figure 8 and Figure 9 show the time–frequency analysis results for train axle box vibration acceleration simulation data under 240 mm and 280 mm wavelength corrugation excitations, respectively, processed by the CEEMDAN–PE-SPWVD algorithm. The identification accuracy for these cases is calculated to be 99.16% and 97.42%, respectively. The results demonstrate that the proposed method can accurately extract wavelength characteristics associated with rail corrugation from the train axle box vibration acceleration signal.

**Figure 10:**
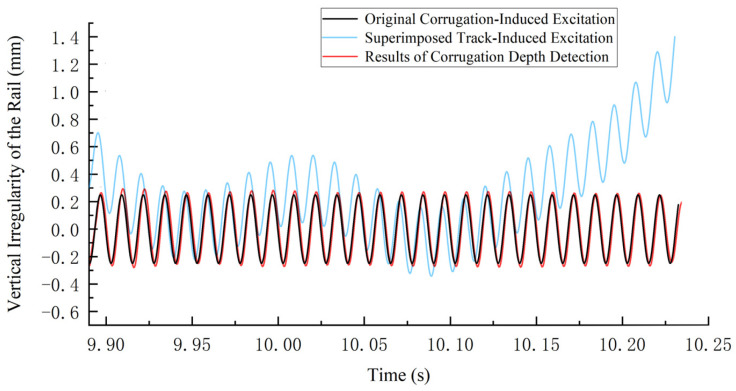
Analysis Results of Corrugation Depth.

Finally, based on the time–frequency analysis results, the filter band range was established, and the filtered IMF_6_ signal was processed using the proposed depth identification method. The results are shown in Figure 10, where the blue curve represents a U.S. Class 5 irregularity track with superimposed corrugation [36]. The depth detection results effectively remove the irregular trend of the track excitation, closely aligning with the trend of the original corrugation excitation, with the waveforms nearly overlapping. The depth characteristics of the corrugation can be determined by calculating the difference between the peaks and troughs of the detected waveform. Analysis of other corrugation simulation data yielded similar results, indicating that this method detects corrugation depth within an acceptable error range.

#### 3.2.2. Method Validation Using Measured Data

**Figure 11:**
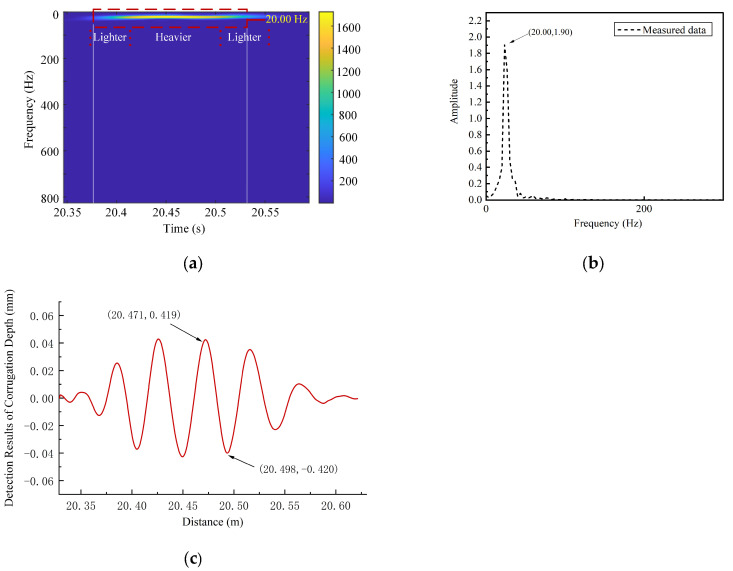
Results for the identified corrugation depth and wavelength for the section: (**a**) SPWVD time–frequency representation; (**b**) Fourier Transform spectrum; (**c**) analysis results for corrugation depth.

**Figure 12:**
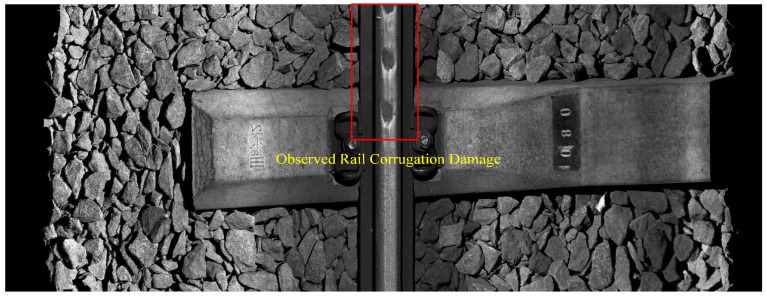
On-site photograph of the identified rail corrugation section.

**Figure 13:**
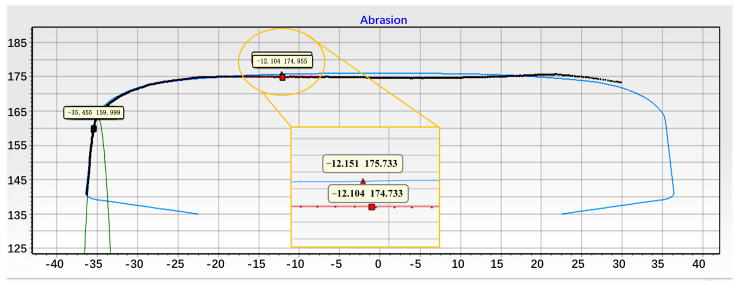
Cross-sectional profile of the rail at the corrugation trough.

The proposed method was applied to process the measured axle box vibration acceleration data collected in this study. Figure 11a illustrates the time–frequency analysis results using the method proposed in this paper, where the strongest energy is observed at a frequency of 20 Hz. Figure 11b presents the Fourier Transform spectrum of the selected modal component, indicating a central vibration frequency of 20.00 Hz, consistent with the time–frequency analysis results. According to Equation (6), the calculated corrugation wavelength is 138.9 mm. Figure 11c shows the results of applying the proposed depth identification method to the measured axle box vibration acceleration data, revealing a clear sinusoidal waveform with an estimated corrugation depth of approximately 0.84 mm. To validate the effectiveness of the identification method, data from the track contour detection device for this section were collected with the assistance of railway personnel. The results are shown in Figure 12 and Figure 13. Figure 12 indicates that the rail corrugation in this section has a strong periodicity, with a wavelength of approximately 142.8 mm, and Figure 13 shows the cross-sectional profile of the rail at the corrugation trough in this section, indicating a corrugation depth of approximately 0.78 mm. A comparison indicates that the CEEMDAN-PE-SPWVD algorithm achieves a wavelength identification accuracy of 97.27% and a depth identification accuracy of 92.97%, demonstrating accurate extraction of corrugation characteristics. Figure 11a reveals that the bright frequency band in the time–frequency analysis results appears exclusively within the 20.37 s–20.57 s time range of the extracted data segment. By analyzing rail surface image data from the track inspection software for this time period, it was confirmed that rail corrugation damage was present exclusively in the sampling frames corresponding to this time range. No significant periodic damage was observed on the rail surface during other time intervals. This suggests that the high-frequency response range of vibration acceleration can effectively locate rail sections with corrugation damage. Furthermore, the section with the highest bright frequency band energy is observed within the 20.42 s–20.52 s time interval. The corrugation results obtained from the track gauge inspection device show that rail corrugation damage is more severe in this section compared to other locations. These findings demonstrate that, under certain conditions, the proposed method can also reflect variations in the severity of corrugation damage across different locations.

### 3.3. Method Comparison

**Figure 14:**
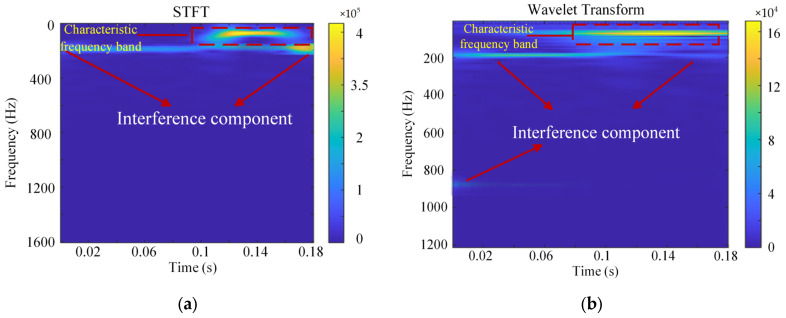
Comparative time–frequency results using different methods: (**a**) STFT results; (**b**) Wavelet Transform results.

To further validate the efficacy of the CEEMDAN-PE-SPWVD method proposed in this study, comparisons were made with the Short-Time Fourier Transform (STFT) and Wavelet Transform methods. STFT is a time–frequency analysis method that processes signals by applying a fixed-length window function [37]. In comparison to STFT, the Wavelet Transform overcomes resolution limitations by providing a time–frequency window that adapts to different frequencies, enabling more flexible signal processing [38]. Figure 14 shows the results of the STFT and Wavelet Transform for vibration acceleration under 200 mm wavelength corrugation excitation. In the experiment, STFT used a window length of 512. From Figure 14a, the width of its characteristic frequency band appears broader compared to that of the proposed method. In Figure 14b, the characteristic frequency bandwidth of the Wavelet Transform is smaller, but it contains many interference frequency components, which significantly affect the identification of rail corrugation. In contrast, the CEEMDAN-PE-SPWVD method proposed in this paper more accurately captures rail corrugation characteristics, demonstrating its superiority and precision in complex signal environments.

## 4. Conclusions

This paper introduces a novel approach for identifying railway rail corrugation utilizing CEEMDAN, PE, and SPWVD techniques. This method employs CEEMDAN decomposition and PE analysis to filter out intrinsic mode components that reflect corrugation excitation. It then applies the SPWVD time–frequency method to analyze the time–frequency characteristics of the selected mode, extracting the bright frequency band range and wavelength information. After filtering components based on the frequency band range, frequency domain integration is used to calculate the corrugation depth. Finally, the algorithm is validated using simulation data from a 3-D finite element wheel–rail model and axle box vibration acceleration data collected under real corrugation excitation conditions. The results show that the proposed method can accurately identify rail corrugation by analyzing axle box vibration acceleration signals, effectively determining its wavelength and depth characteristics.

Compared to other widely used time–frequency methods, the CEEMDAN-PE-SPWVD method extracts corrugation vibration response frequencies more concentrically, without interference frequencies. In the validation with measured axle box vibration acceleration data from a real railway line, the proposed method demonstrates high accuracy in rail corrugation identification, achieving 97.27% accuracy for wavelength characteristics and 92.97% accuracy for depth characteristics, establishing a foundation for further identification of additional corrugation damage features.

## Figures and Tables

**Table 1:** Model Parameters.

	Parameters	Numeric Value
primary suspension	spring mass/kg	12,500
	rigidity/(MN·m^−1^)	2
	damping/(kN·s·m^−1^)	10
fastener system	rigidity/(MN·m^−1^)	24
	damping/(kN·s·m^−1^)	300
wheelset and rail materials	elastic modulus/GPa	205.9
	poisson’s ratio	0.28
	density/(kg·m^−3^)	7790

## Data Availability

All data generated or analyzed during this study are included in this published article.
